# Modeling leukocyte trafficking at the human blood-nerve barrier in vitro and in vivo geared towards targeted molecular therapies for peripheral neuroinflammation

**DOI:** 10.1186/s12974-015-0469-3

**Published:** 2016-01-06

**Authors:** Kelsey M. Greathouse, Steven P. Palladino, Chaoling Dong, Eric S. Helton, Eroboghene E. Ubogu

**Affiliations:** Department of Neurology, Neuromuscular Immunopathology Research Laboratory, Division of Neuromuscular Disease, The University of Alabama at Birmingham, 1825 University Boulevard, Room 1131, Birmingham, AL 35294-0017 USA

**Keywords:** Blood-nerve barrier, Chronic inflammatory demyelinating polyradiculoneuropathy, Experimental autoimmune neuritis, Guillain-Barré syndrome, Intravital microscopy, Leukocyte trafficking, Neuropathic pain, Spontaneous autoimmune peripheral polyneuropathy, Two-photon microscopy

## Abstract

Peripheral neuroinflammation is characterized by hematogenous mononuclear leukocyte infiltration into peripheral nerves. Despite significant clinical knowledge, advancements in molecular biology and progress in developing specific drugs for inflammatory disorders such as rheumatoid arthritis, inflammatory bowel disease, and multiple sclerosis, there are currently no specific therapies that modulate pathogenic peripheral nerve inflammation. Modeling leukocyte trafficking at the blood-nerve barrier using a reliable human in vitro model and potential intravital microscopy techniques in representative animal models guided by human observational data should facilitate the targeted modulation of the complex inflammatory cascade needed to develop safe and efficacious therapeutics for immune-mediated neuropathies and chronic neuropathic pain.

**Electronic supplementary material:**

The online version of this article (doi:10.1186/s12974-015-0469-3) contains supplementary material, which is available to authorized users.

## Background

Peripheral neuropathies are a very common group of disorders that currently affect an estimated 20 million people in the USA alone, and hundreds of millions of patients worldwide. An estimated 20–30 % of cases are cryptogenic or idiopathic, for which no known cause has been determined despite extensive laboratory evaluation. A major consequence of peripheral neuropathies is the development of neuropathic pain, which affects over 1 % of the US population [1, 2]. Treatments for neuropathic pain are non-specific and provide moderate relief at best [3]. Chronic pain syndromes associated with peripheral neuropathies are often medically refractory, with treatments costing the USA economy billions of dollars per year in direct and indirect costs [4].

For acquired neuropathies where it is intuitively expected that pathologic interactions between the systemic circulation and peripheral nerves play an important role in disease initiation or progression or both, aberrant or compromised function of the blood-nerve barrier (BNB) could be key to fundamentally understanding how peripheral neuropathies and neuropathic pain develop. Relatively little is known with regards to the function, significance, and mechanisms of pathogenic alterations at the BNB. When functioning properly, the BNB regulates the movement of solutes, macromolecules, and leukocytes from the circulating blood to the innermost aspects of peripheral nerves and nerve roots, known as the endoneurium [5–7]. Structural alterations such as changes in intercellular junctions associated with leukocyte trafficking and basement membrane thickening/duplication at the BNB have been implicated in the pathogenesis of inflammatory neuropathies and diabetes mellitus [8–12].

Immune-mediated peripheral neuropathies, such as Guillain-Barré syndrome (GBS) and chronic inflammatory demyelinating polyradiculoneuropathy (CIDP), have unknown or incompletely understood triggers; however, the presence of hematogenously derived monocytes/macrophages and T-lymphocytes in affected patient nerve biopsies implies a pathogenic role for leukocyte infiltration across the BNB [9, 13–15]. Similarly, there is evidence supporting mononuclear and polymorphonuclear leukocyte infiltration from the blood circulation into peripheral nerves in the development of chronic neuropathic pain based on animal models [16–20], although direct evidence from patients is lacking.

Neutrophil infiltration has been shown to correlate with increased pain perception in a rat model of partial sciatic nerve transection [20]. Chemokine receptor CCR2+ monocytes/macrophages have also been implicated in chemical-induced inflammatory pain responses and nerve ligation/chronic constriction-induced hypersensitivity to mechanical pain (mechanical allodynia) in rats and mice [21–23], with chemokine receptor CX3CR1+ monocytes associated with chemotherapy (vincristine)-induced allodynia in mice [16]. T-lymphocyte infiltration with subsequent secretion of proinflammatory cytokines such as interleukin (IL)-17A, IL-23, and IL-15 has also been implicated in the pathogenesis of neuropathic pain in the chronic constriction injury model in mice [24, 25], while CD4+ CD25+ Foxp3+ regulatory T-cells have been shown to modulate mechanical allodynia following sciatic nerve chronic constriction injury and experimental autoimmune neuritis in rats [26].

Guided by these observations, we can infer that inhibition of leukocyte infiltration at onset or earlier stages of disease progression could reduce the extent of demyelination and axonal injury in immune-mediated neuropathies and development of chronic pain in inflammatory and traumatic neuropathies, resulting in improved patient outcomes in these disorders. This review article will explore recent advances of in vitro leukocyte trafficking models at the BNB and discuss the potential application of intravital microscopy for in vivo study in animal models. Emerging knowledge from these models based on hypothesis-driven experiments focused on the determinants and signaling pathways for specific leukocyte subpopulation chemoattraction, arrest, transmigration (paracellular or transcellular), and local tissue migration could guide targeted anti-inflammatory drug therapies directed at pathogenic leukocyte trafficking in human peripheral nerve disorders.

## Peripheral nerve anatomy and the blood-nerve barrier

**Fig. 1:**
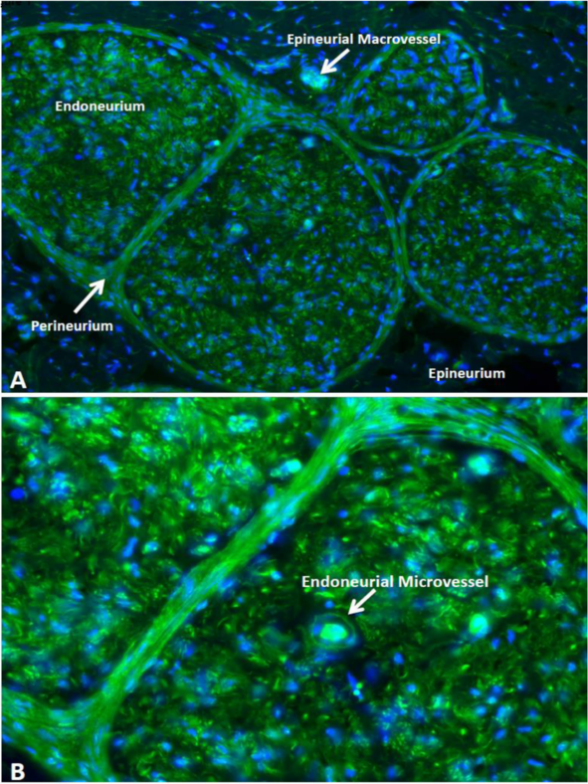
Peripheral nerve structure and vascular supply. A digital photomicrograph of a FITC-conjugated Ulex europaeus agglutinin-1 (*green*) stained frozen section of a human sciatic nerve counterstained with DAPI (*blue*) demonstrates the compartmentalized organization of peripheral nerves, with an epineurial macrovessel depicted in (**a**) and an endoneurial microvessel that forms the blood-nerve barrier depicted in (**b**). Endoneurial microvessels are capillary-like and are derived from epineurial macrovessels that penetrate the perineurium

In order to effectively develop targeted molecular therapies for inflammatory neuropathies, one must first have an understanding of peripheral nerve structure and the BNB. Peripheral nerves consist of an external epineurium, inner perineurium, and innermost endoneurium (Fig. 1). The endoneurium is of critical importance as axons and their associated Schwann cells, which either myelinate segments of single axons or envelope clusters of small axons without myelination, reside within it [7, 27–29]. These myelinated and unmyelinated axons are responsible for signal transduction to and from peripheral nerves. The endoneurial microenvironment is strictly regulated by endoneurial microvascular endothelial cells that form the BNB, as these cells are in direct contact with circulating blood. Perineurial myofibroblasts that form a concentric multilayered barrier around the endoneurium preventing passive influx of epineurial interstitial fluid into the endoneurium or efflux of endoneurial interstitial fluid into the epineurium also play an important role in endoneurial homeostasis [27–31]. The perineurium and the endoneurium it surrounds form a fascicle or nerve bundle, aligned parallel to the longitudinal axis of the nerve. Human peripheral nerves consist of multiple fascicles, with the sciatic nerve (the largest nerve in the body) possessing 50–80 fascicles [32]. This is in contrast to the mouse sciatic nerve that may possess 1–4 fascicles enveloped within a thin epineurial layer [33].

Endoneurial endothelial cells and the innermost layer of perineurial myofibroblasts regulate solute and macromolecular transport, as well as leukocyte entry into the endoneurium due to their specialized intercellular adherens and tight junctions and lack of fenestrae [27, 30, 31, 34, 35]. In contrast, blood vessels within the epineurium do not form a restrictive barrier between the systemic circulation and the epineurial interstitium, as they lack tight junctions and possess fenestrae [28, 29]. Detailed description of the molecular and biophysical properties of the human BNB in vitro and similarities to and differences from the human blood-brain barrier based on comparative studies using immortalized cell lines have been recently published [6, 32, 36]. Human endoneurial endothelial cells that form the BNB are Ulex europaeus agglutinin-and von Willebrand factor-positive, and endocytose acetylated low-density lipoproteins (demonstrating expression of specific lipid scavenger receptors). These cells form intercellular adherens junctions (vascular endothelial cadherin-positive) and electron dense tight junctions (occludin, claudin-5, zona occludens-1 and -2-positive) in vitro and demonstrate high transendothelial electrical resistance and low solute permeability to low and high molecular weight molecules consistent with a restrictive microvascular barrier [6, 32, 37].

These specialized microvascular endothelial cells also express selective mitogen receptors such as vascular endothelium growth factor receptor-2 and glial cell line-derived neurotrophic factor family receptor alpha-1 that drive proliferation, differentiation, response to injury and restoration of barrier function [37, 38]; specific enzymes such as alkaline phosphatase and γ-glutamyl transpeptidase; nutrient transporters such as glucose transporter-1 (GLUT-1), large neutral amino acid transporter-1, and monocarboxylate transporter-1 (MCT-1); and xenobiotic transporters such as p-glycoprotein (also known as multidrug resistance-1a) and organic anion transporter-3 [6, 32, 36]. The retention of essential molecular and biophysical properties expected for a tight junction forming restrictive microvascular barrier in vitro by human endoneurial endothelial cells provides a tool to elucidate key determinants and signaling mechanisms relevant for understanding systemic-neural interactions in health and disease at the BNB.

## Leukocyte trafficking at the blood-nerve barrier

The mechanisms of leukocyte infiltration across the BNB are at the early stages of elucidation, guided by observations in other microvascular barriers. Leukocyte trafficking across the endothelium is a sequential, coordinated process driven by interactions between selectins (such as E- and P-selectin), chemokines, cell adhesion molecules (such as intercellular adhesion molecule-1 (ICAM-1) and vascular cell adhesion molecule-1 [VCAM-1]) expressed on the endothelial cell luminal membrane, and ligands (such as P-selectin glycoprotein-1), chemokine receptors, and integrins expressed by circulating leukocytes. This is referred to as the multi-step paradigm for leukocyte trafficking. Leukocyte rolling, adhesion, and transmigration have been described by pathologists and vascular biologists for many years. However, additional steps have been identified that are crucial for leukocyte migration, dependent on the specific microvascular bed: tethering, slow rolling, adhesion strengthening and spreading, intravascular crawling, and paracellular and transcellular transmigration [39]. Due to the important pathogenic role of hematogenous leukocyte infiltration across the BNB in immune-mediated neuropathies and possibly neuropathic pain as suggested by animal models, it is crucial to have model systems that directly evaluate leukocyte trafficking events at the BNB in real time. In vivo animal and in vitro human models serve as complimentary tools in the quest to elucidate the determinants and signaling pathways of specific leukocyte subpopulation trafficking in peripheral nerves required develop targeted molecular therapies for pathogenic immune-mediated inflammatory neuropathies and possibly neuropathic pain.

## In vitro models of leukocyte trafficking at the blood-nerve barrier

Mechanisms of leukocyte infiltration into the endoneurium are pathologically relevant to immune-mediated neuropathies, such as GBS and CIDP. There are currently no models that allow direct evaluation of leukocyte trafficking in peripheral nerves in vivo. Developing a near-physiological human in vitro BNB has been essential to understanding possible mechanisms of leukocyte-endothelial interactions in vivo [40]. Static and flow-dependent leukocyte trafficking models have provided significant insight into the normal and pathologic mediators of leukocyte subpopulation entry to tissues in different disease states [41, 42]. Direct comparison of flow-dependent (in which endothelial cells are exposed to shear stress) and static blood-brain barrier models showed enhanced endothelial cell differentiation and restrictive barrier characteristics that are further enhanced by culturing endothelial cells on modified hollow chambers with microscopic pores that mimic capillaries [43, 44]. Furthermore, recent studies have demonstrated the importance of flow and shear forces on leukocyte trafficking via induction of selectin-dependent rolling (which is absent in static models), chemokine-dependent integrin activation and arrest, as well as generation of mechanical signals that directly influence biochemical responses of endothelial cells to facilitate leukocyte extravasation such as cytoskeletal alterations during diapedesis [45]. In addition to the importance of flow and shear forces to endothelial cell-leukocyte interactions, there is phenotypic and functional heterogeneity between endothelial cells from different species and tissues, as well as between macrovascular and microvascular endothelial cells from within the same tissue [46–52].

Ultrastructural examination of epineurial macrovessels in peripheral nerves demonstrates lack of electron dense intercellular tight junctions that are present in endoneurial microvessels [28, 29]. Endoneurial microvessels also specifically express alkaline phosphatase [47]. While GLUT-1 and p-glycoprotein expressions are highly conserved in several species (e.g., human, cow, guinea pig, and rat), MCT-1 expression has been demonstrated by human endoneurial endothelial cells (and possibly rabbit), with absent expression in the mouse and rat [32]. Leukocyte trafficking in peripheral nerves predominantly occurs across endoneurial microvessels in immune-mediated peripheral neuropathies such as GBS and CIDP and their rodent animal models, as well as animal models of neuropathic pain, rather than epineurial macrovessels [23, 53]. The preferential trafficking of activated leukocytes across microvascular endothelium (capillaries and post-capillary venules) rather than macrovascular endothelium (e.g., arterioles and venules) during inflammation is evolutionally conserved down to the hagfish dermis [50]. It is therefore paramount to directly study homeostatic leukocyte-BNB endothelial cell interactions relevant for peripheral nerve immune surveillance, as well as pathologic leukocyte trafficking relevant to human peripheral neuroinflammation in vitro using a human BNB cell line incorporating near normal physiological flow and shear forces, and not simply extrapolate from other vascular barriers such as the blood-brain barrier or use endothelial cells from different species or tissues that may differentially express proinflammatory cytokines, chemokines, and cell adhesion molecules.

**Fig. 2:**
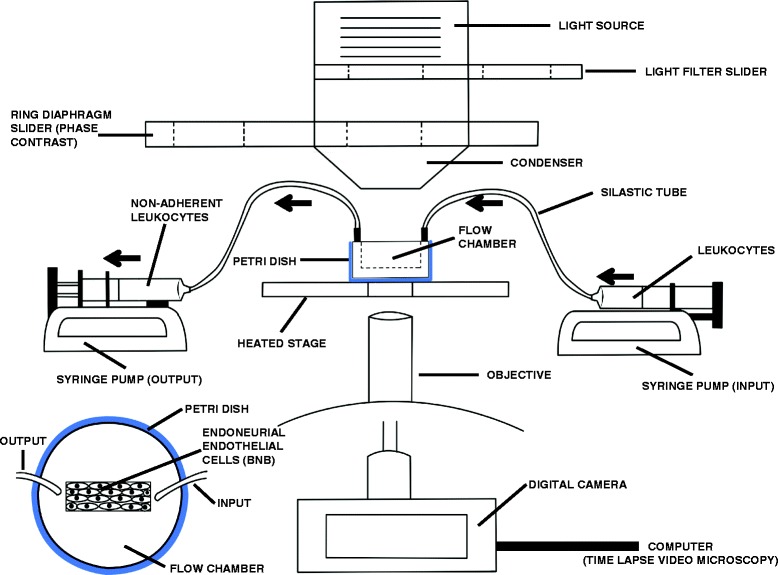
Flow-dependent in vitro blood-nerve barrier model. This illustration depicts a set-up for the flow-dependent in vitro blood-nerve barrier model system that allows direct visualization of human leukocyte trafficking at the BNB in real time using time-lapse video microscopy. Leukocytes are infused at physiologically relevant flow rates and leukocyte-endothelial interactions captured by sequential digital photomicrographs that are merged into videos and analyzed under different experimental conditions. *Black arrows* depict the direction of leukocyte flow

Leukocyte trafficking across the BNB can be studied in real time using a flow-dependent leukocyte trafficking assay that entails a parallel plate flow chamber coupled to time-lapse video microscopy [40, 54–58] (Fig. 2). Primary human endoneurial endothelial cells (pHEndECs) that form the restrictive human BNB have been isolated from the sciatic nerves of decedent patients for culture and used in leukocyte trafficking assay experiments. There is evidence that pHEndECs retain essential molecular and biophysical characteristics of a restrictive microvascular barrier in vitro when cultured in specialized culture medium supplemented with growth factors for up to eight passages [6, 32]. These cells can be seeded on rat-tail collagen-coated CellBIND® petri dishes that facilitate their attachment, proliferation, and contact inhibition. Confluent endothelial cell layers are ready for use in experiments after 5–7 days in culture, forming an in vitro BNB barrier [40, 58].

The in vitro BNB may be treated with physiological concentrations of proinflammatory cytokines (e.g., tissue necrosis factor-α and interferon-γ) up to 48 h prior to experiments to promote leukocyte trafficking without passively altering transendothelial resistance or inducing endothelial cell death [40]. Studying basal leukocyte migration without exogenous cytokine treatment of the BNB may provide insights relevant to the determinants and signaling pathways relevant to peripheral nerve immune surveillance in normal, healthy individuals. This process may be compromised in patients with human immunodeficiency virus infection with peripheral neuropathies. Cytokine-mediated endoneurial endothelial cell activation using concentrations of tissue necrosis factor-α and interferon-γ within the range demonstrated in patients’ sera was associated with de novo or increased expression of chemokines such as CCL2, CXCL2-3, CXCL8, CXCL9, CXCL10, and CXCL11, as well as selectins and cell adhesion molecules such as E-selectin, P-selectin, ICAM-1, VCAM-1, and the alternatively spliced fibronectin variant, connecting segment-1 (a counterligand for leukocyte α_4_ integrin) [40]. These chemokines have been shown to mediate monocyte/macrophage (CCL2), neutrophil (CXCL2-3, CXCL8), and CD4+ T-helper 1 lymphocyte (CXCL9, CXCL10, CXCL11) transmigration [59]. The repertoire of cell adhesion molecules expressed supports leukocyte rolling, adhesion and transmigration in vitro and in vivo based on studies from human brain and non-neural microvessels.

Peripheral blood mononuclear leukocytes (PBMLs) from untreated patients with immune-mediated neuropathies such as GBS or CIDP, or patients with chronic neuropathic pain can be infused across the BNB model at predefined flow rates or shear forces that mimic estimated capillary hemodynamics in peripheral nerves. In this model system, firmly adherent PBMLs become noticeable within seconds and start to form adherent clusters with transmigration after about 5 min, with an increase in the number of attached and transmigrated PBMLs over time, reaching a peak between 20 and 25 min [58]. In order to elucidate the molecular determinants and role(s) of different signaling mechanisms on leukocyte trafficking at the BNB at different stages of the cascade, function neutralizing monoclonal antibodies or small molecular antagonists can be added to the leukocytes or applied to the BNB prior to performing the assay. The effect of leukocyte activation and different stages of a disease on leukocyte trafficking may also be studied using leukocytes obtained from different patients with the same inflammatory neuropathy characterized for activation markers by flow cytometry and leukocytes obtained from the same patient at disease onset, peak severity, and during the recovery phases, respectively.

Based on our review of hundreds of videos generated in the Neuromuscular Immunopathology Research Laboratory using PBMLs from healthy donors and untreated patients with immune-mediated neuropathies, our initial observations of leukocyte trafficking at the human BNB in vitro are as follows: as hypothesized by the multi-step paradigm of leukocyte extravasation at vascular beds, leukocyte trafficking at the human BNB consists of rolling, attachment with or without post-adhesion locomotion to sites presumed to express high concentrations of chemoattractant molecules (haptotaxis), firm adhesion concentrated at intercellular membranes, and transmigration via the paracellular route. Endothelial cell cytokine activation is a stronger stimulus for leukocyte trafficking than systemic leukocyte activation that occurs in GBS. GBS patient-derived mononuclear leukocytes preferentially adhere to the BNB in vitro (Additional file 1: Video 1), dependent on α_M_ integrin-ICAM-1 mediated signaling (Additional file 1: Video 2), with CD14^+^ monocytes being the most prevalent adherent leukocyte subpopulation [40]. Monocytes/macrophages are the most prevalent leukocyte subpopulation observed within the endoneurium in peripheral nerve biopsies of human GBS and its representative animal model, experimental autoimmune neuritis (EAN) [60, 61], further validating the utility of the flow-dependent in vitro BNB model to understand pathogenic leukocyte trafficking in peripheral nerves in vivo.

By using pHEndECs as well as patient-derived PBMLs with estimates of capillary flow rates and alterations in the endoneurial microenvironment in vivo, this in vitro human BNB model currently provides the most physiologic means to study human peripheral nerve inflammation pathogenesis as a tool to guide the discovery and potential efficacy of specific molecular-based target drugs to treat immune-mediated neuropathies such as GBS and CIDP. Our previous work demonstrated the differential expression α_M_ integrin (CD11b) on GBS patient leukocyte subpopulations and its critical role in leukocyte transmigration via ICAM-1 at the BNB using this model [40], suggesting potential efficacy in GBS that is currently being tested in animal models. There are no leukocyte trafficking assays published using other human primary or immortalized endoneurial endothelial cells.Static leukocyte assays using CCR2-expressing human acute monocytic leukemia cell line and primary or immortalized rat endoneurial endothelial cells [62, 63] provide some insights relevant to understanding peripheral neuroinflammation but neither utilize patient-derived leukocytes nor a human BNB model, limiting translational potential due to likely interspecies differences and the inadequacies of static transmigration assays.

Limitations of the described flow-dependent human in vitro BNB model include the use of a parallel plate system rather than a hollow, capillary-like, microtube chamber that allows more accurate application of physiological shear forces; the absence of an abluminal compartment to apply chemoattractant molecules and collect transmigrated leukocytes for downstream analyses; suspension of patient-derived leukocytes in medium with less viscosity than circulating blood, potential alterations (e.g., downregulation in chemokine receptors) with cryopreserved patient-derived leukocytes ex vivo, potential changes in BNB endothelial biology with in vitro culture; and unknown effects of endothelial cell culture without potentially supportive Schwann cells and pericytes. Despite these limitations, this model provides an avenue to determine whether endothelial cytokine activation or leukocyte activation state influences leukocyte rolling velocities and probability of firm leukocyte arrest, determine which chemokine ligand-receptor pairs are crucial for leukocyte subpopulation arrest and integrin activation, as well as subsequent transmigration at the BNB, determine the differential roles of ICAM-1, VCAM-1, and fibronectin connecting segment-1 in specific leukocyte subpopulation adhesion as well as the signaling mechanisms involved in leukocyte docking at intercellular junctions and paracellular migration at the human BNB relevant to normal immune surveillance and pathogenic neuroinflammation.

## In vivo models of leukocyte trafficking at the blood-nerve barrier

**Fig. 3:**
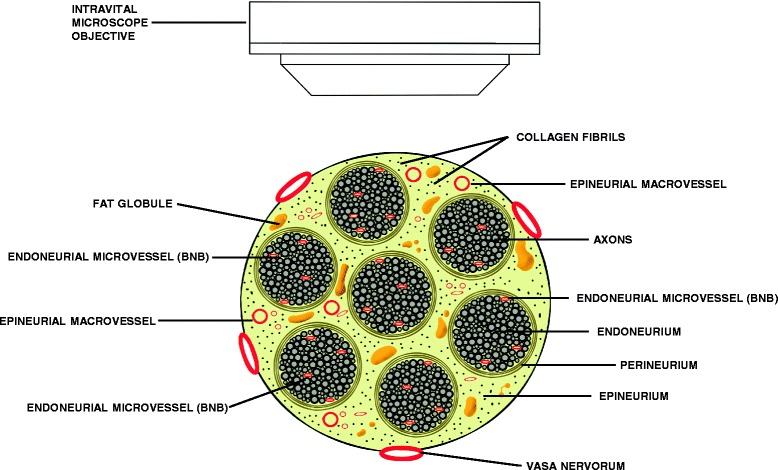
Intravital microscopy of human peripheral nerve. The challenges of visualizing leukocyte trafficking in endoneurial microvessels that form the blood-nerve barrier by intravital microscopy due to the multilayered organization of the human peripheral nerve are demonstrated in this illustration. Barriers that impede clear detection of leukocytes within these microvessels include axons and surrounding connective tissue within the endoneurium, the concentric and multilayered perineurium, and the longitudinally aligned collagen fibers and fat globules with the epineurium. The extrinsic vasa nervorum and epineurial macrovessels should be avoided as these blood vessels lack tight junctions and possess fenestrations

There are currently no models that permit direct assessment of leukocyte trafficking across the BNB in real time in vivo. However, there are various microscopic techniques that allow us to visualize leukocyte trafficking in vivo that may advance our understanding of the multi-step paradigm and provide technical considerations that could guide peripheral nerve leukocyte trafficking studies in the future. The structural organization of peripheral nerves provides a challenge to directly viewing leukocyte trafficking at the BNB in vivo as these low-density microvessels are located within the innermost endoneurium surrounded by axons and loose arrays of collagen fibers aligned in parallel with these microvessels. Furthermore, the endoneurium is surrounded by the multilayered perineurium (forming a fascicle or nerve bundle), and these nerve bundles are embedded within the epineurium consisting of longitudinal arrays of collagen fibers. Superficial extrinsic peripheral nerve blood vessels (i.e., the vasa nervorum) and epineurial macrovessels are theoretically easier to visualize; however, these vessels lack tight junctions and possess fenestrations and as a consequence are not part of the restrictive BNB (Fig. 3). Visualizing leukocyte trafficking in small rodent peripheral nerves is conceptually more likely than in higher mammals due to a thinner epineurium and perineurium, as well as fewer fascicles in anatomically similar nerves.

Intravital microscopy (IVM) techniques have been used for decades with bright field transillumination to help scientists understand the biophysical conditions under which leukocytes are exposed to in microvessels [64–66]. Thus, IVM provides a tool to acquire quantitative, qualitative, and dynamic insights into cell biology and endothelial-leukocyte interactions relevant to understanding the pathophysiology of inflammation in different disease states [67]. Over the last decade, technological advances have been applied to IVM allowing dynamic visualization of leukocyte trafficking within deep tissues. Such advances include application of single, and subsequently multibeam two-photon microscopy, dual or multicolor two-photon imaging, and epi-illuminescence fluorescent microscopy facilitated by injecting specific dyes into rodents intravenously or intraperitoneally. These allow for the visualization and tracking of leukocytes in the brain in vivo with improved temporal and spatial resolution [68–72].

Brightfield IVM is a basic technique used to observe leukocyte trafficking; however, ideal tissues must be translucent and cannot contain any internal structures that scatter or absorb light. This is not the case in peripheral nerves with the multilayered organization that consists of longitudinal arrays of collagen fibrils within the epineurium and less compact arrays within the endoneurium in which the microvessels reside with myelinated and unmyelinated axons. This along with the fact that at physiological flow rates, leukocytes may be obscured by rapidly moving red blood cells make this an impractical model to observe leukocyte infiltration at the BNB. Furthermore, only cells that are clearly rolling or stationary can be detected by this technique [68]. More advanced techniques have emerged that allow in situ leukocyte trafficking observation including two-photon microscopy which can be synchronized with second harmonic generation signals derived from the extracellular matrix [73–75]. The end result is the visualization of immune cell migration and cell-cell communication in the interstitium of solid organs and the brain via intracranial windows [75–77].

A limitation of single beam two-photon IVM is the requirement of higher laser power to enhance sensitivity and detection speed which results in sample degradation. Multibeam two-photon IVM using a multifocal scan head that splits a laser beam into a line of 64 foci with charge-coupled device field detector allows real-time sample illumination at full power. This technique has been shown visualize tissues down to penetration depths of 200 μm at an image quality similar to surface detection after applying depth-correction point of spread function [70]. However, single two-photon IVM with laser detection using photomultipliers generated better images at depths >300 μm due to superior signal-to-noise ratio.

Epi-luminescence fluorescent IVM can be applied to track leukocyte trafficking in vivo by injecting specific fluorophores or fluorescent dyes that facilitate the detection of microvessels. Using Rhodamine 6G to visualize real-time leukocyte-endothelial interactions in the brain, intraperitoneal administration was demonstrated to be as effective as the standard intravenous injection route [72]. However, the intense excitation of light that may be necessary for visualization can cause phototoxic effects that may manifest as enhanced leukocyte adhesion [68]. As opposed to injecting a dye to illuminate microvessels, leukocyte populations can be tagged in vitro before injection into an animal model. Once injected into the recipient animal, the cells are easily detectable because they are the only fluorescent cells in circulation [78]. In addition to studying inflammation, these techniques may be used to study brain tumors in living animals (typically employing two different fluorescent tags). Transgenic mouse models with targeted cell populations labelled with fluorescent proteins of different colors have been used for six-color two-photon IVM of malignant brain tumors generating 5D images in which individual cell populations and components of the extracellular matrix can be separated by spectral deconvolution [71].

IVM and two-photon microscopy have been employed to visual leukocyte-endothelial interactions and elucidate determinants of leukocyte trafficking in mouse models of central nervous system inflammation such as experimental autoimmune encephalomyelitis (an animal model of multiple sclerosis) [79–83], endotoxin-induced inflammation [67, 84–86], herpes simplex virus-1 encephalitis [87] and cryptococcal meningoencephalitis [88]; controlled cortical impact injury model of traumatic brain injury [89] and in healthy living brain slices [75]. Epifluorescence IVM is more suitable to investigate leukocyte-endothelial interactions, particularly tethering and rolling, signal transduction pathways controlling integrin activation, slow rolling, arrest, and adhesion strengthening in the central nervous system vessels, while multiphoton microscopy is more suitable for the investigation of intraluminal crawling, transmigration, and motility inside the CNS parenchyma [90].

Although it has been debated whether IVM adequately visualizes deeper-residing cerebral post-capillary venules that form the blood-brain barrier in addition to superficial vessels that do not, these studies have been useful in determining the role of proinflammatory signals: microglial and endothelial activation, Toll-like receptors, specific selectins and their counterligands, leukocyte integrins and endothelial cell adhesion molecules, and chemokines and chemokine receptors; as well as anti-inflammatory signals such as cannabinoid receptor-2 and annexin A1 interacting with its receptor FRR2/ALX in trafficking of total leukocyte or leukocyte subpopulation such as T-cells (naïve and autoaggressive, antigen-specific), neutrophils or dendritic cells in the central nervous system inflammation [67, 75, 79–89].

Hopefully, the advances made in IVM and two-photon microscopic techniques to visualize leukocyte trafficking in the central nervous system will guide approaches needed to study leukocyte trafficking across the BNB in the near future. These approaches if applicable to the mouse sciatic nerve may aid to elucidate the molecular determinants and signaling mechanisms relevant to T-cell immune surveillance in healthy nerves, monocyte, T-cell, and neutrophil trafficking into the endoneurium at different stages of acute and chronic immune-mediated demyelinating neuritis or peripheral neuropathy following nerve crush injury, as well as aid to determine the leukocyte subpopulations and molecular signals essential for peripheral nerve recovery following inflammation and trauma. These observations could translate to human peripheral nerves due to similarities in neural-immune responses between mice and humans.

Since IVM approaches have not been developed for peripheral nerves, leukocyte trafficking at the BNB in vivo is currently studied indirectly using animal models of peripheral nerve inflammation. Animal models of acute and chronic demyelinating neuritis such as EAN, chronic-relapsing EAN, and spontaneous autoimmune peripheral polyneuropathy (SAPP) have been used to aid decipher relevant mediators and signaling mechanisms of pathogenic leukocyte trafficking that should be preferably guided by in situ observational data from peripheral nerves obtained from affected patients [53]. Despite the fact that these models recapitulate some essential features of the human disorder they mimic, a major drawback is that these animal models do not allow real-time assessment of leukocyte trafficking at the BNB and may utilize signaling mechanisms irrelevant to human GBS, CIDP, or chronic neuropathic pain. Nonetheless, pharmacologic intervention studies in these models using drugs efficacious at nanomolar or low micromolar concentration may provide in vivo proof of principle necessary for early stage clinical trials. Drugs should be administered after clearly discernable neuromuscular dysfunction at time periods reflective of human disease presentation, thereby increasing translational relevance. It is hoped that insights obtained from these animal models will not only further our understanding of leukocyte trafficking in peripheral nerves but also provide specific molecular targets for treating pathogenic leukocyte trafficking in immune-mediated polyneuropathies and possibly chronic neuropathic pain.

## Conclusions

Currently available treatments for immune-mediated neuropathies and chronic neuropathic pain are partly efficacious or focus on symptom control. Recent advances in cell and molecular biology as well as medicinal chemistry and pharmacology should drive the development of specific therapies for these disorders. Although other pathologic aspects of peripheral nerve inflammation and nerve recovery after injury are important considerations, knowledge of aberrant leukocyte infiltration at the BNB has potential to halt the deleterious effects of hematogenously derived leukocytes in these disorders. In a similar vein, care would be required not to impede the trafficking of regulatory leukocyte subpopulations into the peripheral nervous system and be aware of the potential potent systemic immunosuppressive properties specific anti-inflammatory drugs could possess. Using in vitro and in vivo models designed to mimic essential aspects of human peripheral nerve inflammation guided by observational human peripheral nerve data, knowledge obtained will help further elucidate important signaling pathways and hopefully guide the development of more specific and efficacious treatments for pathologic peripheral nerve inflammation.

## Additional file

Additional file 1:
**GBS patient mononuclear leukocyte trafficking at the blood-nerve barrier** ***in vitro*** **.** A video of merged digital photomicrographs in real time acquired between 10 and 15 min following infusion of an untreated GBS (AIDP variant) leukocytes across a cytokine stimulated monolayer of human primary endoneurial endothelial cells demonstrates leukocyte rolling, firm adhesion (phase bright), and some transmigration (phase dark) (Video 1). Following incubation of the same patient’s leukocytes with 500 ng/mL function neutralizing mouse anti-human α_M_ integrin antibody demonstrates significant reduction in adhesion and transmigration in this model system (Video 2), providing evidence for the role of α_M_ integrin signaling in pathogenic mononuclear cell trafficking at the blood-nerve barrier in vitro, with therapeutic implications for GBS.

## Abbreviations

BNB: blood-nerve barrier
CIDP: chronic inflammatory demyelinating polyradiculoneuropathy
EAN: experimental autoimmune neuritis
GBS: Guillain-Barré syndrome
GLUT-1: glucose transporter-1
ICAM-1: intercellular adhesion molecule-1
IL: interleukin
IVM: intravital microscopy
MCT-1: monocarboxylate transporter-1
PBMLs: peripheral blood mononuclear leukocytes
pHEndECs: primary human endoneurial endothelial cells
SAPP: spontaneous autoimmune peripheral polyneuropathy
VCAM-1: vascular cell adhesion molecule-1
